# Genomic and clinical factors associated with drug-resistant tuberculosis

**DOI:** 10.3389/fpubh.2026.1960921

**Published:** 2026-10-21

**Authors:** Karthikeyan Sundaram, Sridhar Rathinam, Ramalingam Bethunaickan, Uma Devi Ranganathan, Madhavan Dhanapal, Venkataraman Prabhu

**Affiliations:** 1 Centre for Herbal Pharmacology and Environmental Sustainability, Chettinad Hospital and Research Institute, Chettinad Academy of Research and Education, Kelambakkam, Kanchipuram, Tamilnadu, India; 2 Chettinad Hospital and Research Institute, Chettinad Academy of Research and Education, Kanchipuram, Tamilnadu, India; 3 Department of Immunology, ICMR- National Institute for Research in Tuberculosis, Chennai, Tamilnadu, India; 4 Faculty of Medical Research, Academy of Scientific and Innovative Research (AcSIR), Ghaziabad, India; 5 Division of Medical Research, SRM Medical College Hospital and Research Centre, Kattankulathur, Chengalpattu, Tamilnadu, India

**Keywords:** antitubercular drugs, drug-resistant, mycobacteria, rifampicin-resistance, tuberculosis

## Abstract

**Background:**

Drug-resistant tuberculosis is a significant global health issue caused by mutations in *Mycobacterium tuberculosis*, the infectious agent of the disease. WHO’s End TB Strategy mandates drug susceptibility testing for rifampicin in all confirmed tuberculosis cases and fluoroquinolone testing for rifampicin-resistant cases.

**Objective:**

The review aims to analyse *Mycobacterium tuberculosis* genome mutations; Clinical factors linked to drug resistance. Major thematic findings: The mutant otsB1 trehalose-6-phosphate phosphatase shows rifampicin resistance in hypoxic conditions. Pyruvate dehydrogenase E2 is vital for RNI resistance, host cell death, and *Mycobacterium tuberculosis* growth. Individuals with drug-susceptible tuberculosis showed higher plasma levels of inducible nitric oxide synthase. NO and iNOS expression can be influenced by factors other than mycobacterial infection. Activated macrophages convert L-arginine to NO via TNF-α and IFN-γ. Serum L-arginine levels were measured in individuals with pulmonary tuberculosis, their household contacts, and healthy controls. PPE51, PE15, and PE20 improve drug resistance by transporting metal ions and absorbing nutrients. In diagnosis, the CRISPR-guided mutagenic DNA polymerase identifies changes in drug-resistant *Mycobacterium tuberculosis*. *Mycobacterium smegmatis*, recognized for its rapid growth, and the slow-growing *Mycobacterium tuberculosis* were evaluated using CRISPR-guided DNA polymerase. Also, machine learning and whole-genome sequencing predicted isoniazid, Rifampicin, and multidrug-resistant tuberculosis with 90% precision. The BDQ DILI network can offer valuable support on monitor the adverse events of the Anti-tuberculosis therapy.

**Conclusion:**

Whole genome sequencing, tNGS, and deletion-targeted multiplex PCR effectively detect mutations. Studying PE/PPE genes offers a key chance to drug-resistant tuberculosis research and improve tuberculosis treatment outcomes.

## 1 Introduction

Tuberculosis (TB) remains a major public health issue globally, with an estimated 10.7 million cases in 2024 (95% uncertainty range: 9.9–11.5 million) (1). In this context, a major challenge in eradicating the global tuberculosis epidemic by 2030 is the rise of drug-resistant tuberculosis (DR-TB), especially rifampicin-resistant tuberculosis (RR-TB); also, multidrug-resistant tuberculosis (MDR-TB) that resists both rifampicin (RIF) and isoniazid (INH) (2). In 2021, approximately 450,000 new cases of RR-TB were reported. The WHO’s End TB Strategy states that all bacteriologically confirmed TB patients must have drug susceptibility testing for rifampicin, and all RR-TB patients should undergo DST for fluoroquinolones at a minimum. In 2021, 70% of patients with diagnosed tuberculosis were tested for rifampicin resistance. Also, isoniazid resistance was linked to six compensatory *ahpC* mutations by regression. They coexist with resistance mutations to reduce their fitness cost compared to the wild-type, *KatG* neutralizes ROS and activates isoniazid. The reduced activity of *katG* due to INH resistance mutations is sometimes compensated by promoter region alterations that increase *ahpC* production. It restores the bacterium’s reactive oxygen species resistance (3, 4).

The global prevalence of isoniazid resistance without rifampicin resistance in TB patients was 9.5% (95% CI 8.0–11.0) in 2014. The global averages were 8.1% (6.5–9.7) for newly treated cases and 14.0% (11.6–16.3) for previously treated cases. In another research, 4.8% of anticipated TB cases were multidrug-resistant, suggesting that most isoniazid-resistant patients are mono- or polydrug-resistant (5). Also, resistance to ethambutol, especially from mutations in the *embB* codon 306, is a significant risk factor for RR-TB and MDR-TB. Besides that, molecular genotyping for MTB in the TB resistance profile often analyses a narrow genomic segment, limiting its ability to differentiate effectively. Thus, whole genome sequencing (WGS) reveals sub-lineage diversity, drug resistance, host response, transmissibility, and toxicity through genomic variation (6–8). Similarly, the Mycobrowser database is an aid to analysis; however, it shows approximately 4,000 annotated open reading frames in the MTB genome. Currently, 1803 protein-coding genes remain unidentified, including 68 putative transmembrane proteins. *Rv2617c*, found only in the *M. tuberculosis* complex, belongs to this final category of proteins. *Rv2617c* is a protein that interacts with P36 (Mb3850, Erp, or *PirG*), a virulence factor of MTB and *Mycobacterium bovis* (9). In this context, analysis of lineage in MTB is crucial; notably, in a recent study in eastern Sudan, L3 strains are the primary cause of pulmonary TB (Delhi/CAS). L3 strains are vital to TB epidemiology in eastern Sudan due to treatment resistance and recent spread. 73.4% of culture-positive pulmonary TB patients had L3 strains (10). Another study using standard genotyping methods found that at least 40% of the Central Asia spoligotype family was present in central and eastern Sudan. Analysis showed Sudan as a key African L3 hotspot (11–14). Therefore, this review objective is to analyse MTB genome mutations; Clinical factors linked to drug resistance.

## 2 Search methodology

This review examines the factors linked to tuberculosis resistance, genetic mutations related to anti-tubercular drugs, and advanced techniques for identifying these mutations. The subsequent keywords employed include rifampicin-resistant tuberculosis, isoniazid-resistant tuberculosis, factors associated with TB resistance, Anti-Tuberculosis Drugs (ATD)-related gene mutations, multidrug-resistant TB, and diagnostic platforms for identifying TB resistance. This review employed many scholarly databases, such as PubMed/Medline, Science Direct, Springer Link, Scopus, and the WHO report. A cumulative total of 20,165 studies published in English were examined, and we included publication years spanning from 2015 to 2025. Studies were excluded due to irrelevant titles, non-tuberculous diseases, reviews, mini-reviews, duplicates, inadequate data, antiquated literature, and other linguistic literature. In this literature search, 95 studies were assessed concerning the parameters linked to DR-TB. In addition, we examined 9 study datasets derived from the notable findings of genetic biomarkers associated with resistance and diagnostic platforms.

## 3 Factors linked to tuberculosis resistance

Antibiotic resistance and treatment failure are linked to changes in specific gene sequences, particularly alterations in the resR gene. Genetic alterations in the MTB genome are more prevalent in drug-resistant bacteria; however, these changes do not directly lead to drug resistance. Additional bacterial genomic markers linked to therapeutic failure in drug-sensitive MTB isolates require examination, as drug resistance significantly affects treatment effectiveness (15, 16). The initial resistance of anti-TB drugs diminished the effectiveness of nitric oxide (NO) in eliminating MTB. Both *M. intracellulare* and *M. tuberculosis* CDC1551 exhibit resistance to acidified nitrite; however, the precise mechanism by which nitric oxide influences mycobacteria remain ambiguous. Additionally, a recent study revealed that patients with drug-susceptible TB exhibited elevated plasma levels of inducible nitric oxide synthase when compared to those with drug-resistant strains. NO and iNOS expression could stem from influences that extend beyond MTB infection. Macrophages stimulated by TNF-α and IFN-γ produce nitric oxide from L-arginine. This study evaluated serum L-arginine levels among patients with pulmonary tuberculosis, their household contacts, and healthy control subjects. Patients with PTB exhibited elevated serum L-arginine levels in comparison to both household contacts and healthy controls. Infection improves L-arginine levels, resulting in heightened iNOS activation and nitric oxide production. This finding may be connected to a metabolite derived from amines that is linked to lung pathology (17–19).

Additionally, ferritin was elevated in alveolar macrophages, not in interstitial macrophages, aligning with high and low intracellular MTB growth. However, there is a significant link between TB patients’ blood ferritin levels, bacillary burden, and lung damage. Thus, the intracellular MTB may benefit from more ferritin. In addition, MTB increases ferritin breakdown through nuclear receptor coactivator 4 (NCOA4), increasing iron bioavailability and bacterial growth. Hence, studies on ferritinophagy in macrophages revealed that MTB hijacks of host ferritin metabolism for replication and its role in human TB infection (20). The diminished oxygen response may be influenced by the inactive *PhoP* and the irregular *dosS/dosR* regulon of MTB H37Ra. Although the expression of the *PhoP* and *dosS/dosR* regulon, the MDR and XDR mycobacteria exhibited thicker cell walls compared to susceptible strains; nonetheless, the cell membrane of H37Rv became thicker over 18 months of hypoxic conditions. Furthermore, the equivalent TOL synthesis from MTB H37Rv and H37Ra in hypoxic conditions indicates that the *dosS/dosR* and *PhoP* regulons are not interconnected. Consequently, routes lacking *PhoP* may persist (21).

Noticeably, the strains with the Beijing genotype had a higher cluster. This result aligns with earlier studies and indicates that the Beijing genotype may have increased transmissibility (22, 23). In contrast, a study in England found no link between transmissibility among household contacts and the Beijing genotype strains. Another study also noted similar findings, indicating that DR-TB patients produce larger aerosol volumes of culturable MTB. This investigation found that 20.21% (19/94) of clustered isolates were isoniazid-resistant. However, 11% of isoniazid-resistant tuberculosis patients treated with the standard regimen experienced treatment failure, compared to 2% of drug-susceptible patients (2, 24–27). Recurrence significantly increased RR-TB in older patients compared to TB patients with and without recurrence to identify risk factors. However, reducing the length of treatment raises the possibility of relapse (28). Also, HIV status significantly influenced RR-TB recurrence. Guerra-Assunção et al. found that being HIV positive had a greater impact on reinfection than on relapse. In contrast to earlier studies, 50% (3/6) of HIV-positive tuberculosis patients experienced relapse, while the other 50% were reinfected (29).

Monocyte-derived interstitial macrophages (IM)-derived MTB increases carboxymycobactin-producing genes in two clusters, *mbt1* and *mbt2*. The genes associated with the iron-regulated ABC transporter *IrtAB*, which helps absorb Fe+3-carboxymycobactin, also increased (30). Iron-controlled protein *PPE37* is essential for heme-iron absorption. In MTB IMs, *PPE37* expression was significantly elevated, suggesting that this iron acquisition method may be essential for MTB’s *in vivo* survival (31).

Furthermore, metabolomic analysis of MTB strains revealed alkanes and D-glycero-L-mannoheptonic acid, a glycolipid surfactant that absorbs alkanes. MTB alkane metabolism and function remain unclear. The MTB *AlkX* gene is located after *alkB-rubAB*. The MTB *alkB-rubAB* gene is repressed by the *TetR* regulator *AlkX* from Dietzia DQ12-45-1b, as stated by Liang et al. This study found that palmitic acid hindered *AlkX* DNA-binding. They discovered a feedback mechanism that promotes positive product outcomes in the degradation of alkanes producing long-chain fatty acids (32, 33).

### 3.1 Mutation on mycobacterial genes links to resistance

Resistance to streptomycin and ethambutol (EMB) in MDR-TB has significantly risen. A recent study found that 62.3% of 130 MDR-TB isolates from 48 retreated and 52 treatment-naïve patients were resistant to EMB, while 72.3% showed resistance to streptomycin (SM) (34). However, a notable study examined EMB resistance, highlighting its significance. Mutations in the *embB* gene, which encodes arabinosyltransferase, are a major cause of EMB resistance (35). EMB resistance was associated with mutations in *embB* gene codons 306, 406, and 497, as well as the *ndh gene* R268H mutation. Ethionamide and isoniazid co-resistance is due to the R268H mutation. This investigation identified an F285L mutation in an isolate that is sensitive to EMB (36).

Recent research found 30 new mutations. Most involved adding or removing nucleotides of different lengths. Mutations occur between nucleotide locations 21–521. At previously mentioned nucleotide positions, 176C ≥ A, 232G ≥ C, 412 T ≥ G, and 425C ≥ G substitutional new mutations occurred. Also, study found that the 176C ≥ T mutation is sensitive to Pyrazinamide (PZA) at 100 μg/mL. The clinical isolate’s PZase activity was positive for the new mutation 176C ≥ A, indicating PZA susceptibility. Mutations 232G ≥ T, 232G ≥ A, 412 T ≥ C, and 425C ≥ T increase PZA resistance, while newly identified mutations 232G ≥ C, 412 T ≥ G, and 425C ≥ G decrease PZase activity, indicating resistance in clinical isolates. PZA resistance also arises from mutations in *rpsA, clpC1, panD*, and efflux pump genes, alongside *pncA* mutations (35, 37).

Rifampicin-resistant strains with the low fitness cost mutation rpoB S450L were linked to more drug-resistant-conferring mutations, and the number of drug-resistant-conferring mutations rose even more when the strain also comprised compensatory mutations. It has already been discovered that compensated strains exhibit more mutations that give drug resistance in different settings (38–40). Other reasons indicate that confounding is unlikely to affect study observations. This study also extended the multivariable regression analysis for genetic clustering to a stringent analysis and found that using only the initial sampled isolate from each cluster yielded identical results. Second, the number of drug-resistant mutations should correlate with the length of time a genotype has been in circulation and evolving. However, this does not explain why S450L and non-S450L rpoB variations are associated with drug-resistant mutations (39, 41).

Seven PE/PPE genes (*PPE51, PPE13, PE15, PE20, PE23, PE7,* and *PPE10*) were recently identified that may be linked to drug resistance. Proteins like PPE51, PE15, and PE20 help metal ions cross the outer membrane and absorb nutrients (42, 43). The *PPE51–PE19* pair may absorb glycerol, glucose, and phosphate as a porin-like assembly. PE/PPE pairs transport nutrients across the outer membrane, but their role in drug resistance remains unclear. Wang et al. found that removing *PPE51* from MTB made it resistant to small molecules by blocking growth-inhibiting chemicals. PPE51 underwent positive selection when bacteria acquired multidrug resistance mutations in katG and rpoB. Of the 48 changes in PPE51, 43 were frameshift insertions/deletions or premature termination mutations, suggesting that selection pressure results in the impairment of PPE51 functionality. Prior to the *PPE51* mutations, multiple strains exhibited *gyrA, rrs*, and *mmpR5* mutations. These changes indicate advanced drug resistance, with nonsynonymous *PPE51* mutations prominent in isolates resistant to six to eight anti-TB drugs. Thus, the absence of *PPE51* may lead to resistance against new drugs like bedaquiline (BDQ) or linezolid (LZD), typically used when first- and second-line treatments fail (43–45). A mutation in the ESX-5 type VII secreted protein EsxW-Thr2Ala may increase Beijing lineage strains in Vietnamese and other host populations. Other changes may also impact MTB transmission dynamics, alongside antibiotic resistance mutations (46, 47).

### 3.2 Functions of various resistance genes

PE/PPE proteins are crucial for host-pathogen interactions and bacterial physiology. Bacterial physiology includes drug resistance, metabolism, enzymatic activity, and nutrient absorption (44). Their interactions with Toll-like receptors (TLRs) influence phagosome maturation, antigen presentation, autophagy, cell death regulation, and immune responses in both innate and adaptive systems. PE/PPE proteins are rich in T-cell and B-cell epitopes, inducing strong immune responses, making them promising subunit vaccine candidates and diagnostic tools (48, 49).

The sensor kinases *DosS* (*Rv3132c*), *DosT* (*Rv2027c*), and response regulator *DosR* (*Rv3133c*) let MTB adapt to anaerobic conditions for latent infection. Oxygen deficiency and increased NO, CO, and ROS activate the *DosR* gene. Carbon monoxide, hypoxia, and NO activate the *DosR*-dependent regulon, which maintains bacterial growth under situations of stress (50). The *PhoP* response regulator and sensor kinase *PhoQ* are one of the most conserved two-component signal transduction systems in bacteria. *PhoP/PhoQ* mediates signal transduction. Sensing host environmental cues including Mg2+, low pH, antimicrobial peptides, and osmotic pressure, it affects bacterial environmental tolerance, virulence, adhesion, and invasion genes. *PhoP/PhoQ* system-induced signal composition feedback. The active state of *PhoP* phosphorylation influences gene transcription and expression, which affects bacterial stability (51).

MTB development involves seven Wbl proteins (WhiB1–7). The *whiB1* gene encodes a DNA-binding protein with a nitric oxide-sensitive iron–sulfur cluster [4Fe–4S], consisting of three α-helices in a four-helix bundle. *WhiB3* regulates lipid anabolism in MTB to maintain intrabacterial redox balance (52). Also, *AlkB* is a predicted hydroxylase for alkanes, and overexpression of the AlkBRubAB system in the *alkX* mutant enhanced MTB survival in both activated and resting macrophages (32, 33). Furthermore, the progression of tuberculosis is intricately linked to various genes and their mutations at specific sites. Consequently, the investigation of specific gene variations is crucial. Moreover, the recent emergence of testing methodologies may aid in identifying specific gene abnormalities.

## 4 Advanced diagnostic methods to distinguish TB resistance

CRISPR-directed DNA polymerase may allow *E. coli* and *Saccharomyces cerevisiae* to precisely diversify genes of interest. This method uses a bacterium with a Cas9 nickase (nCas9)-DNA polymerase (PolA) fusion protein to create a single sgRNA for the target gene. The Cas9 nickase finds and cuts a gene target. CRISPR-guided DNA polymerase machinery was tested in slow-growing MTB and fast-growing *M. smegmatis*, revealing new resistance mutants with drug-targeted gene inhibitor binding sites (53–55). MTB DNA polymerase A was used to develop an error-prone DNA polymerase using protein structural alignment. In an 80-bp editing window, CAMPER system inserted random substitution mutations using MTB’s error-prone DNA polymerase A and the highly compatible XCas9 nickase, which identifies NGN as the protospacer adjacent motif (PAM) CAMPER system created site-directed mutagenesis in *M. smegmatis* and MTB to find drug-targeted gene inhibitor binding site-resistant mutants (54, 56). Additionally, CAMPER can improve the analysis of protein-drug interactions. This method swiftly identifies drug-resistant gene reactions to protein activity.

Furthermore, high nxCas9-PolA3M^TB^ expression reduced bacterial growth, indicating mild proteotoxicity. Mycobacteria exhibit first-generation Cas9spy proteotoxicity. Second-generation Cas9sth1 is safe for gene knockdown and knockout in mycobacteria. The unique PAM sequences of Cas9sth1 stop it from targeting specific sequences. Studying innovative Cas9 with high adaptability and low toxicity in mycobacteria may enhance this tool. nxCas9-PolA3M^TB^ had an editing window of ∼ 80 bp. PolA’s DNA polymerase domain flexibility also affects mutations. Thus, future studies on generating DNA polymerase domain mutations to increase mutation frequency and window duration (53, 57, 58). The conserved transmembrane protein *Rv2434c* is not critical for the *in vitro* growth of MTB in nutrient-abundant environments, as per a recent Mycobrowser study. This suggests it might aid in cellular function but is not essential for life under these circumstances. Nonetheless, the alterations at the 317th codon of the protein induce resistance to AM, CM, MFX, OFX, and PTO. The processes underlying these interactions are not well understood (59).

Machine learning methodologies forecasted resistance to INH, RIF, and MDR-TB at 90% accuracy by utilizing comprehensive mutation and locus information. Nonetheless, this model omitted 10% of the resistance. First-line treatments (EMB, 20%; PZA, 60%) and second-line treatments (AMK, CAP 20–25%; ETH, 35%; CYS, 55%) exhibited enhanced genotypic–phenotypic discordance in the GBT-ALL model, suggesting unidentified genetic factors. Epigenetics, heteroresistance, overexpression of efflux pumps, drug susceptibility test breakpoints, and laboratory DST inaccuracies can provide assistance (60, 61). In a study on ML classifiers, the Mykrobe predictor demonstrated a notable enhancement in the F1-score for all four primary TB medications and one secondary treatment relative to sophisticated rule-based predictions. The F1 score serves as the principal criterion for assessing classification models. The four methodologies are evaluated based on diverse standards. The F1 score reconciles accuracy and sensitivity via the harmonic mean. The research incorporated the geometric mean of sensitivity and specificity (G-mean) because the F1-score lacked True Negatives. These metrics are more challenging to analyse in conditions of class disparity. F1-scores are essential for analysing and mitigating bias in imbalanced datasets since they reconcile precision and recall. Three machine learning methods surpassed the rule-based Mykrobe prediction in F1-score across all four first-line medications and one second-line therapy, with the 1D CNN classifier attaining the maximum efficacy (62). Histone deacetylase 2 (HDAC2), linked to transcriptional response variations to MTB infection in monocyte-derived macrophages from persistently negative tuberculin skin test (PTST-) and non-PTST- individuals, may be disrupted by a binding motif in ZEB2 (63). The macrophage is essential to the innate immune response and significantly influences MTB pathogenesis from recognition to destruction. Genetic diversity in macrophage response may influence resistance to MTB infection. Enhancer histone marks and DNaseI-hypersensitivity sites in various immune cell types show numerous SNPs with strong associations in Sample 1, indicating active transcription of ZEB2 in these cells (64). Notably, various diagnostic methods are now being assessed for their efficacy in detecting TB resistance (Table 1).

**Table 1:** Selected bacterial and host genetic factors associated with tuberculosis drug resistance and treatment response.

Study	Resistance markers	outcomes	Treatment/ diagnostic platform	Reference
Dai Y et al., 2023	*TRIM21 E3,*	RIM21 mRNA and protein levels were examined in Mtb-infected THP-1 macrophages treated with p38, JNK, NF-κB, or AKT1 inhibitors to determine Mtb’s impact on TRIM21 signaling pathways SB202190 and AKTi substantially decreased TRIM21 induction. The p38 and AKT1 signaling pathways increase macrophage TRIM21 expression after Mtb infection.	RT-qPCR/ Immunoblot analysis	(20)
Chen et al., 2023	*otsB1, Rv3168, cobN, dlaT, metA Rv0648,and Rv1248c*	The clinical prediction model based solely on host risk characteristics exhibited an AUC of 0.70, compared to 0.58 for GWAS-detected bacterial mutations. When host variables and GWAS gene mutations were added, the model’s AUC for poor treatment outcomes increased from 0.70 to 0.74 with 0.85 sensitivity and 0.55 specificity.	Fixed single nucleotide polymorphisms (SNPs) and genome-wide association studies (GWAS)	(15)
Gan M et al., 2025	*PE36, lipX, PPE40, PE15, PPE37 - PE36 affected 598 isolates with 1.67 pNpS, 105 NS, and 28 SY mutations. LipX affected 584 isolates (1.43 pNpS, NS: SY = 84:23)*.	Early lipX codon 78, 88, and 92 halt mutations. The PPE40 mutant has 0.42 pNpS, 165 NS, and 167 SY mutations. ESX-3 substrate PE15 binds to PPE20 for Ca^2^ + transport, while PPE31 may be implicated in Mg^2^ + uptake. PPE36 and PPE37 pNpS are higher in drug-resistant isolates.	WGS analysis	(43)

## 5 Future research and strategy to diminish TB resistance

The TB resistance becomes a global threat due to the long-term trends of MDR-TB/XDR-TB incidence in different countries and regions, exploring disease burden and control efficacy differences by development level (65). It shows where wealthier nations have failed to manage drug-resistant TB and highlights low-income countries’ ongoing issues. Other factors also affect these changes. Poor anti-TB medicine use, early identification and treatment issues, and lack of regulation promote resistance (66–68). Specifically, increase active TB screening among high-risk immigrants, improve cross-border notification mechanisms, and use digital case monitoring tools to manage TB. Regional surveillance, real-time pathogen and resistance monitoring, and genomic surveillance to track high-risk clones should also be prioritized (69).

In future research, in silico tools are promising on TB mutation identification; however, β-lactam function and synthetic lethality depend on drug-resistant gene pairs like *whiB4, blaI, atpH, embB, katG,* and *furA*. Mutations and changes in gene expression of *whiB4* impact TB treatment response, determined in single-gene knockouts or overexpression (70). In addition, the work shows that *blaI* and its downstream targets *atpH* and *sigC* influence collateral vulnerability to conventional drug resistance but do not directly impact *blaC* β-lactamase gene transcription. A significant evolutionary link between drug-resistant and β-lactam genes is indicated by their functional similarities, including resistance to MTB cell wall-targeting drugs like isoniazid and ethambutol (71, 72).

On the other hand, normal infections raise stress levels, potentially leading to more mutations. However, studies anticipate higher mutation frequency test rates due to increased DNA damage and repair. High mutation rates can select hyper-mutators, potentially increasing drug resistance and chronic illness progression (73). SNPs reveal mutations in genes related to the immune response of host microbes. *ALKBH5* and *FTO* gene variants affect MTB immune response and PTB risk. Notably, the diverse populations include Chinese demographic showed a strong link to obesity through the *FTO* gene rs9939609 (74). Obesity, like other forms of malnutrition, reduces leukocytes and cell-mediated immunity. Malfunctioning immune systems increase susceptibility to MTB and coxsackievirus. *FTO* gene variation may influence immune-related viral diseases like PTB through obesity-related immunocompetence (75). Li et al. found a strong correlation between the lncRNA NEAT1 rs3825071 variation and sputum smear positivity in PTB patients. Similarly, there is a substantial correlation between sputum smear positivity and the rs8400 AA genotype, A allele, and rs9913266 A allele frequencies (76). Thus, the hierarchical control of these SNPs may help design PTB treatment options.

Additionally, future research on two-component system genes is essential for understanding MTB mutations. Specifically, the *PhoP* mutations occurred in all MTB mutants selected for beta-lactam resistance. *PhoPR* regulates complex lipid biosynthesis genes in two components, impacting MTB cell wall composition and virulence. System response regulators have *PhoP*’s C-terminal effector and N-terminal regulatory domains. This investigation showed that AMX/CLAR I (Lys167Glu) and MEM/CLAR isolates (Phe185Ser) have C-terminal amino acid changes, while II and III mutants (Thr99Ala) exhibit regulatory domain modifications (77). Thus, the enhanced beta-lactamase action in these 4 mutants may outcome from mutations in the transcription process.

The management of DR-TB includes screening the adverse events of the Anti-tubercular therapy (ATT). In the BDQ-DILI network, ten core genes with the highest connections were selected from the 76 probable BDQ-DILI targets. A key finding was that BDQ-related DILI targeted the PI3K/Akt pathway (78). In cancer, inflammation, and metabolic disorders, the PI3K/Akt pathway has been intensively studied for its involvement in cell survival, proliferation, and metabolism. The PI3K/AKT pathway controls cellular responses to medicines and stress. Akt is phosphorylated and activated by many growth hormones and cytokines. Akt stimulates cell growth and proliferation while preventing apoptosis. By incorrectly boosting or inhibiting the PI3K/Akt signaling pathway, hepatotoxic drugs can damage the liver (79, 80).

## 6 Limitation of mutation detection in TB resistance

Tuberculosis resistance detection possesses substantial limitations; however, the limited coverage of smaller healthcare facilities is likely due to a lack of defined laboratory protocols, skilled personnel, and necessary equipment for molecular drug-susceptibility testing (mDST) (81). In this context, deletion-targeted multiplex polymerase chain reaction (DTM-PCR) is faster and easier than RFLP but less effective than mycobacterial interspersed repetitive unit-variable-number tandem repeat (MIRU-VNTR) analysis. Drug-resistant gene sequencing is crucial since WGS is expensive and requires extensive data processing (Table 2). Whereas Gen-TB predicts INH and RFP resistance poorly compared to the WHO mutation database and other WGS methods. Gen-TB only used INH-resistant mutations katG_S315N, S315T, and fabG1_C-15 T. The WHO database of mutations includes resistant *inhA* gene variants (inhA_G154A, T770C, and C777T), which may explain its higher sensitivity (83.17%) than Gen-TB (81.19%). Most RFP-resistant strains (95% or greater) had 81 bp *rpoB* gene alterations (82–85). Also, He et al. correlated with the earlier findings that the *rpoB* gene variants S431G, Q432L, D435Y, H445D, H445Q, and L452P are to be absent in Gen-TB, explaining its limited RFB resistance sensitivity (82).

**Table 2:** Various studies on antitubercular drug resistant genes detected from advanced diagnostic tool.

Study	Resistance markers	Outcomes	Treatment/ diagnostic platform	Reference
Zhan J et al., 2024	katG S315T, rpoB S450L, L430P, katG S315T, rpsL K43R, embB M306V, and rrs A1401G.	KatG S315T—88.46% (77.93%) isoniazid-resistant (89/115). The most common mutations, embB M306V and rpsL K43R, made EMB and SM 60 and 68.46% resistant. Capreomycin, kanamycin, and amikacin were 16.92%, 16.15%, and 19.23% resistant. AK, CM, and KM possessed A1401G mutants.	WGS analysis	(34)
Kulkarni et al., 2025	*mmpS5_c.-74G > T. Tier 1 bedaquiline genes* (*atpE, mmpS5, mmpL5, Rv0678, and pepQ.* katG*_*c.12). A > G, *inhA_*c.-777C > T	lower sensitivity compared to regression for 13 out of 15 drugs (0.081 to 10.2 pp). 12/15 medications showed PPV greater than regression and SOLO specificity (0.011–3.70 pp., 0.10–12.7 pp). The 15 medications had regression 70.2%, SOLO + GR 69.9%, and SOLO 69.2% F1 (harmonic mean of sensitivity and PPV). More than specificity (−1.0 pp) and PPV (−1.6 pp), sensitivity (+3.2 pp) deviates from regression.	SOLO and regression model data sets	(4)
Nguyen QH et al., 2025	*GyrA and B, rpoB, A, and C. KatG*	In MDR *M. tuberculosis* strains, the mutations rpoB_Ser450Leu, katG_Ser315Thr, inhA–15(C–T), gyrA_Asp94Gly, and rrs_1401(A–G) were frequently observed in significant proportions of 46.3%, 76.4%, 16.2%, 8.9%, and 11.4%, respectively.	24-loci MIRU-VNTR profiles and spoligotyping	(46)
Wang et al., 2022	*katG, rpoB, and rpsL*	Patients under 65 clustered more than those 65 or older (AOR = 3.11, 95% CI: 1.76–5.49). Residents clustered more with Beijing genotype strains, with an odds ratio of 2.43 and a 95% confidence interval of 1.18 to 4.99. Isoniazid-resistant individuals clustered more (AOR = 2.36, 95% CI: 1.15–4.88).	SNPs	(28)
He G et al., 2024	*inhA, rpoB*	Variants of the resistant inhA gene (inhA_G154A, T770C, and C777T) could account for its greater sensitivity of 83.17% compared to Gen-TB’s 81.19%. Over 95% of RFP-resistant bacteria possess a mutated 81 bp rpoB gene. Gen-TB exhibits a limited sensitivity to RFB resistance, attributed to the lack of mutations in the rpoB gene, specifically S431G, Q432L, D435Y, H445D, H445Q, and L452.	WGS analysis	(82)
Goig GA et al., 2023	*rpoB*	Most common in rpoB S450L (730 [62·5%]), followed by D435V (7·1%), H445Y (5·5%), L452P (4·2%), L430P (3·9%), H445D (3·8%), and D435Y (3,300 (91.7%)) – Rifampicin. Compensated strains were lineage 2, 27 (8.3%) lineage 4. Lineage 2 – mutation in rpoB S450L.	WGS analysis	(39)

Furthermore, identifying the specific mechanisms by which PTB induced interstitial lung abnormalities is unclear and needs to be evaluated; though, studies suggest that improper immune system regulation may be a primary cause. An imbalance between Th1 and Th2 cytokines may disrupt humoral and cell-mediated immune responses, increasing lung interstitium inflammation that may lead to pulmonary fibrosis (86, 87). Additionally, advances in post-TB lung disease rehabilitation should improve outcomes for RR-TB survivors and reduce burden relative to existing estimates. Superior diagnoses and treatments for RR-TB may reduce the illness burden compared to drug-susceptible disease (88).

## 7 Discussion

In this review, we examined multiple factors and various genomic mutation linked to the DR-TB (Figure 1). Non-adherence to ATT treatment, which increases disease recurrence. Consequently, the specific infection’s adverse effects lead to medication drop-out. However, environmental and social variables strongly affect adherence. Effective patient-physician communication in DOT systems is crucial for constant evaluation and adherence. Also, utilize low-income travel subsidies for adherence support (89).

**Figure 1:**
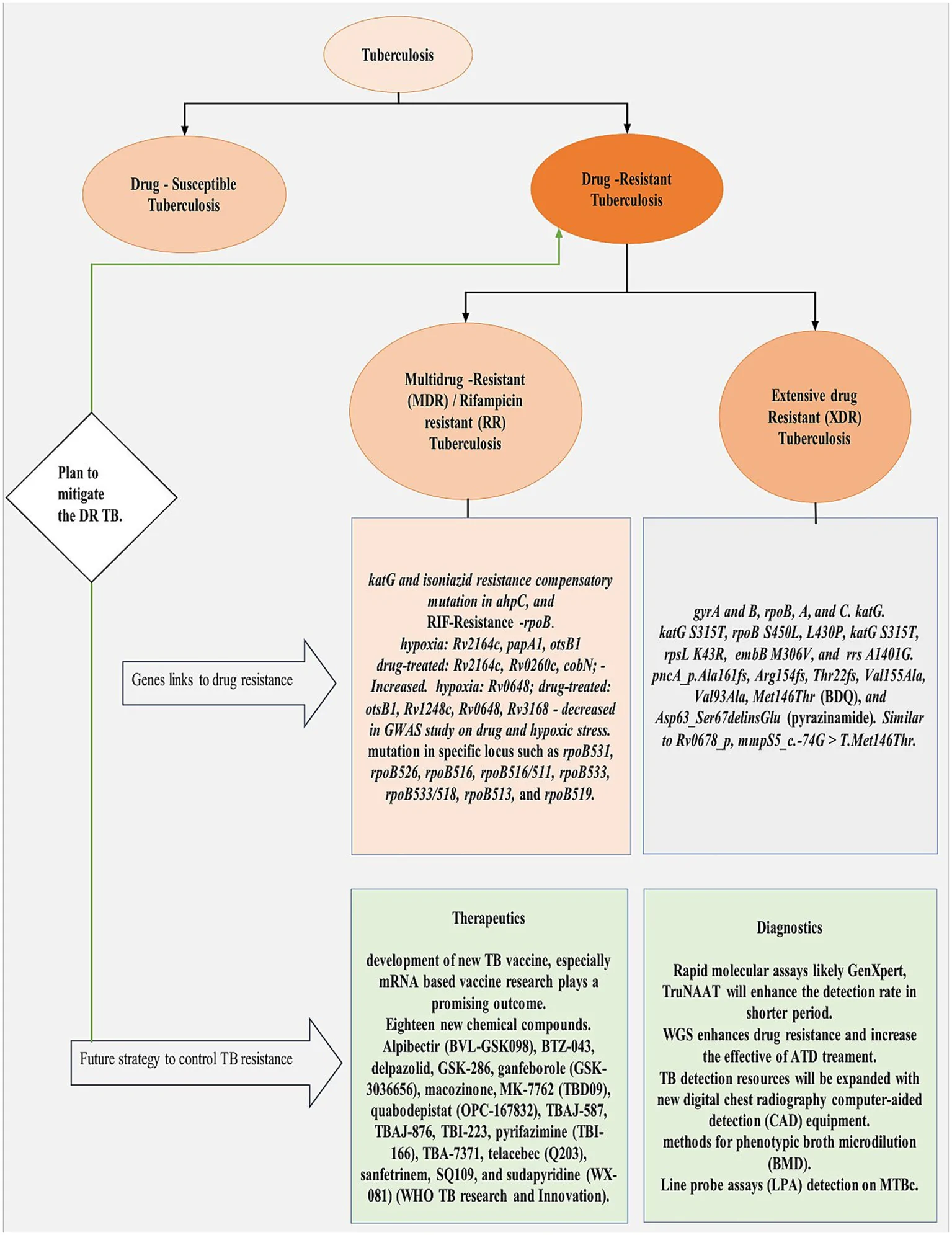
Illustrated the future strategy to control the TB infection with current development of TB therapeutics and diagnostics effectiveness (2, 8, 19, 80).

Tuberculosis resistance is significantly associated with the prevalence of PE/PPE proteins. Predominantly, these proteins are mostly seen in slow-growing or pathogenic mycobacteria. Their function has long been unknown while making up 10% of MTB’s genetic coding capacity. MTB’s metabolic relationship with the host is being altered by mutational pressures impacting feeding and metal ion transport PE/PPE genes. However, the type VII secretion system of (T7SS) ESX-3 substrate PE15 transports Ca^2+^ and forms a functional compound with PPE20. PPE31 may absorb Mg^2+^ (42, 43). In this context, drug-resistant isolates also had higher pNpS values for PPE36 and PPE37, requirements for heme iron uptake, indicating continued adaptation to iron availability. Besides, surface-exposed membrane proteins PPE36/PE22 and PPE62 interact with heme and are necessary for the MTB model to use heme and hemoglobin, showing that these routes converge at the cell surface. An unknown mechanism provides heme to PPE36/PE22 and PPE62 from hemoglobin. This experiment showed that MTB proliferation stopped when PPE36 was completely removed, although PPE62 only slightly reduced heme use, demonstrating PPE36’s importance. Using heme, MTB needs the substrate-binding protein DppA. Since this study have not ruled out the idea that DppA could transfer heme to an unrelated permease, making MTB’s heme acquisition an uncertain. Thus, metal transport and drug resistance may be linked by cross-resistance mechanisms or regulatory pathways (43, 90, 91).

Moreover, the advantage of regular WGS is predicting drug resistance and enabling tailored drug selection, enhancing patient care. Bacteria culture yields sufficient DNA for genome sequencing. Delayed MTB development postpones treatment for 2–4 weeks and increases costs. Drug-resistant TB patients require prompt and effective treatment. Besides, the constraints of WGS pose a significant issue, mostly owing to cost rather than convenience in small laboratory settings; yet the benefits of WGS are considerable. However, WGS can identify drug-resistant mutations in minimal MTB forms that phenotypic Drug Susceptibility Testing (pDST) cannot detect. Unrecognized minority variations with drug resistance may be selected without enough therapy, causing therapeutic failure and clinical relapse. Despite having high-confidence drug-resistant mutations, 6.8% of strains had genotypic DST sensitivity, compared to 1% previously reported. Minimal variants with drug resistance-related mutations exceeding 3% were found in 3.5% (55/1,586) of pDST-susceptible strains. It is commonly known that the precision and sensitivity of pDST can be lacking (31, 37, 92).

The GenoScreen Deeplex and Oxford Nanopore Technologies (ONT) TB Drug Resistance Test are used to identify drug-resistant TB. Rebecca E. Colman and colleagues studied the MycTB test in a multicenter trial using two tNGS tests. Both tests show over 95% sensitivity and specificity for identifying isoniazid and rifampicin resistance. Fluoroquinolones are essential second-line TB drugs, showing 98.8% ONT specificity, 99.8% GenoScreen specificity, and sensitivities of 95.7 and 92.7%. Second-line TB drug resistance testing was unreliable. Furthermore, limitations of the proprietary tNGS tests and unclear lab methods hindered the identification of therapeutic ineffectiveness. In bioinformatics, genomic analysis reveals only known mutations. Probes and databases have not fully identified all drug resistance mechanisms, including the all-oral BPaLM regimen for MDR/RR TB. PCR probe design and gene sequencing research must adjust to drug resistance correlations (92, 93).

*FLOT1*, a lipid raft-linked membrane protein, controls *Mycobacterium marinum* and host-pathogen immunity. *FLOT1* may have a similar role in MTB-macrophage interactions. *FLOT1* affects signaling and cell migration pathways, hence changing expression may impact the host immunological response to MTB. The g2g-L2 human risk allele may influence more than *FLOT1*. After MTB infection, immune-related genes such PPP1R18, an adapter protein in RAPK signaling, and HLA-E, which can present MTB peptides, exhibit differential expression. The intronic mutation should change these genes (94, 95).

Therefore, the resistance to various ATT associated with tuberculosis requires greater focus on clinical manifestations, patients’ treatment adherence, and reducing diagnostic delays; however, advanced diagnostic tools are used to detect genetic mutations to improve accuracy, which can lead to more effective treatment strategies and better patient outcomes. However, this approach will incur higher costs compared to current methods. Moreover, recent advancements in molecular-based analysis have enabled the reduction of diagnostic time constraints on the detection of TB resistance, which will enhance adherence to anti-tubercular treatment (Figure 2). While molecular-based diagnoses are high in sensitivity and specificity, cost is slightly a concern, but it could be more manageable than conventional diagnostic methods, such as AFB, which is less sensitive and specific, and MTB culture, which requires a significant amount of time to detect.

**Figure 2:**
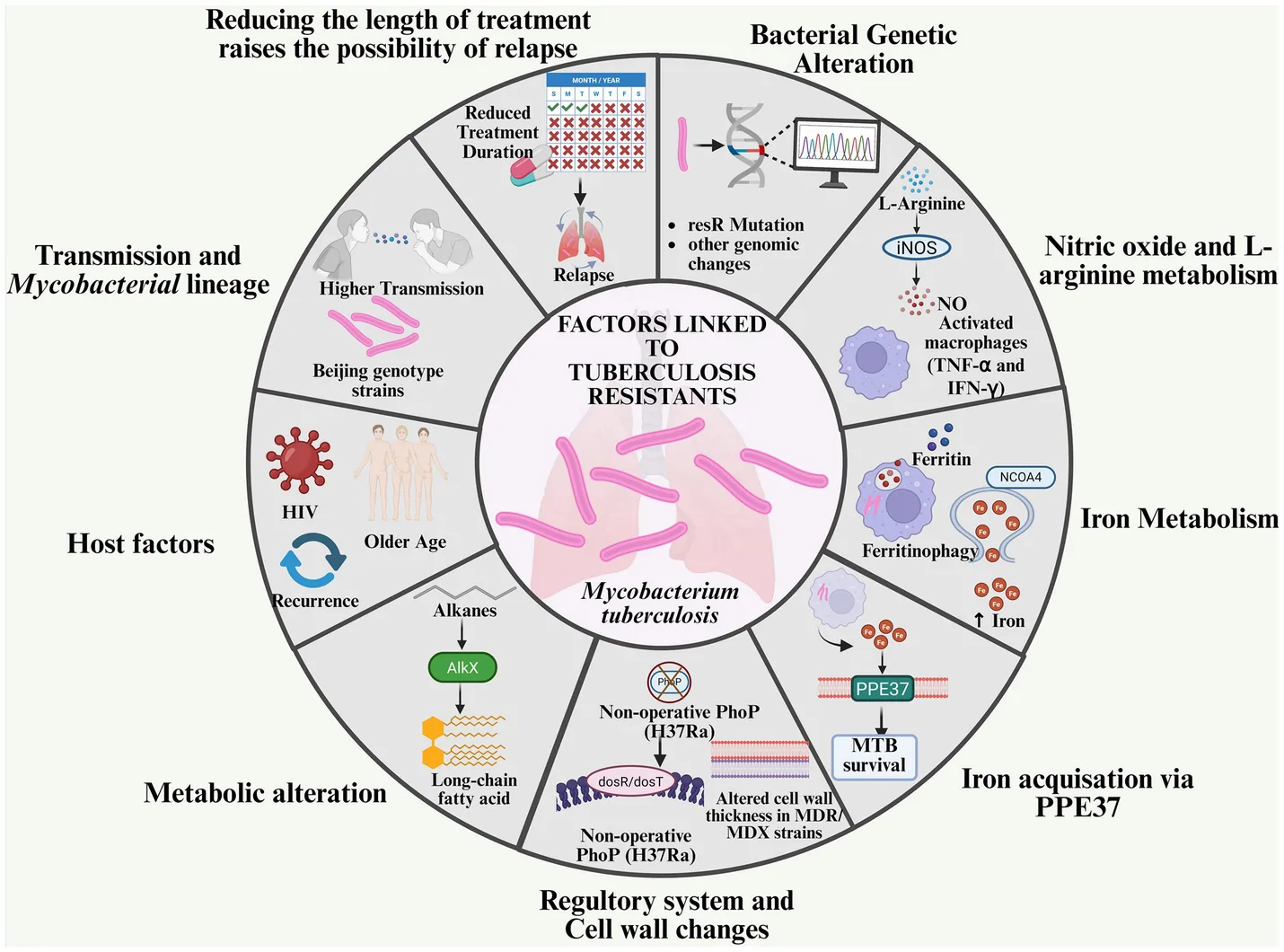
Schematic representation of the major factors linked to drug resistance in *Mycobacterium tuberculosis*. These factors include mycobacterial genetic alterations, nitric oxide and L-arginine metabolism, iron homeostasis and acquisition, regulatory and cell-wall adaptations, metabolic reprogramming, host-related factors, bacterial lineage and transmission, and antibiotic exposure or treatment-related factors. These include: (i) genetic changes in mycobacteria, such as *resR* mutations and other genomic alterations; (ii) metabolism of nitric oxide and L-arginine, including inducible nitric oxide synthase (iNOS), macrophage activation, TNF-α, and IFN-γ responses; (iii) iron metabolism, including ferritinophagy, NCOA4-mediated ferritin degradation, and intracellular iron accumulation; (iv) iron uptake and cell-wall alterations, including non-operative *PhoP* (H37Ra), the *DosR/DosT* regulatory system, altered cell-wall thickness, and phenotypic changes linked to multidrug-resistant/extensively drug-resistant strains; (v) cell-wall and regulatory changes, such as non-operative *PhoP* (H37Ra), the *DosR/DosT* regulatory system, altered cell-wall thickness, and phenotypic changes linked to multidrug-resistant/extensively drug-resistant strains; (vii) host-related factors, such as aging, HIV infection, and disease recurrence; (viii) transmission and mycobacterial lineage, such as the Beijing subtype strains lineage and increased transmission or clustering; and (ix) antibiotic exposure and treatment-related factors, including rifamycin overdose, treatment failure, and associated lung injury, which may contribute to selection and persistence of resistant organisms.

## 8 Conclusion

This review comprehensively analysed mutations in various genes associated with TB resistance and evaluated patient treatment with antitubercular drugs based on the identification of specific gene mutations. Addressing the management of DR-TB presents numerous challenges; however, the implementation of advanced diagnostic techniques like WGS, tNGS, and DTM-PCR has the potential to significantly enhance treatment outcomes. Recent studies indicate that PE_PPE genes play a significant role in disease progression. Therefore, examining these genes across various clinical strains may provide insights into the resistance patterns.
